# Long-Term Outcomes in Children with Steroid-Resistant Nephrotic Syndrome Treated with Calcineurin Inhibitors

**DOI:** 10.3389/fped.2015.00104

**Published:** 2015-11-27

**Authors:** Nathan T. Beins, Katherine M. Dell

**Affiliations:** ^1^Division of Pediatric Nephrology, Children’s Mercy Hospital, Kansas City, MO, USA; ^2^Center for Pediatric Nephrology, Cleveland Clinic Foundation, Cleveland, OH, USA

**Keywords:** nephrotic syndrome, calcineurin inhibitor, steroid resistant, AKI, FSGS

## Abstract

**Background:**

Steroid-resistant nephrotic syndrome (SRNS) is an important cause of chronic kidney disease (CKD) in children that often progresses to end-stage renal disease (ESRD). Calcineurin inhibitors (CNIs) have been shown to be effective in inducing short-term remission in some patients with SRNS. However, there are little data examining their long-term impact on ESRD progression rates.

**Methods:**

We performed a retrospective chart review of all patients treated for SRNS with CNIs at our institution from 1995 to 2013. Data collected including demographics, initial response to medical therapy, number of relapses, progression to ESRD, and treatment complications.

**Results:**

A total of 16 patients met inclusion criteria with a mean follow-up of 6.6 years (range 0.6–17.6 years). Histopathological diagnoses were focal segmental glomerulosclerosis (8), mesangial proliferative glomerulonephritis (4), IgM nephropathy (3), and minimal change disease (1). Three patients (18.8%) were unresponsive to CNIs while the remaining 13 (81.2%) achieved remission with CNI therapy. Six patients (37.5%) progressed to ESRD during the study period, three of whom did so after initially responding to CNI therapy. Renal survival rates were 87, 71, and 57% at 2, 5, and 10 years, respectively. Non-Caucasian ethnicity was associated with progression to ESRD. Finally, a higher number of acute kidney injury (AKI) episodes were associated with a lower final estimated glomerular filtration rate.

**Discussion:**

Despite the majority of SRNS patients initially responding to CNI therapy, a significant percentage still progressed to ESRD despite achieving short-term remission. Recurrent episodes of AKI may be associated with progression of CKD in patients with SRNS.

## Introduction

Nephrotic syndrome is a rare clinical syndrome consisting of high grade proteinuria, hyperlipidemia, hypoalbuminemia, and edema. Nephrotic syndrome affects ~2–7 children per 100,000 and affects all ages and ethnic backgrounds (1–4). Prior studies have demonstrated that ~80% of children diagnosed with nephrotic syndrome will respond to steroid therapy (5). However, there is recent evidence that the incidence of initial steroid resistance is increasing (6). Previous long-term follow-up studies have demonstrated favorable prognosis if remission is achieved with steroids (7, 8).

The majority of patients with steroid-resistant nephrotic syndrome (SRNS) will have focal segmental glomerulosclerosis (FSGS) found on biopsy (2, 5). Historical studies examining SRNS, specifically caused by FSGS, provided evidence that >50% of children who do not respond to initial steroid therapy would progress to end-stage renal disease (ESRD) within 3 years (9, 10). Due to the unfavorable prognosis of SRNS, numerous therapies have been utilized in an attempt to achieve remission, including cyclosporine, tacrolimus, cyclophosphamide, and rituximab. The most predominant therapies utilized are the calcineurin inhibitors (CNIs), cyclosporine, and/or tacrolimus, which are the current recommended first line therapy for SRNS per the 2012 Kidney Diseases Improving Global Outcomes (KDIGO) Guidelines (11). Numerous studies have demonstrated their efficacy in achieving short-term remission (12–14). While several studies have examined long-term outcomes in SRNS (15–18), there are many limitations to these results. The goal of the current study was to examine the long-term (>5 years) follow-up of SRNS patients treated with CNIs at a single institution, with specific attention paid to those who initially showed good response to CNIs.

## Materials and Methods

A retrospective chart review was performed of all patients with SRNS treated with CNIs from January 1995 through August 2013 at Rainbow Babies and Children’s Hospital. Patients were identified via ICD-9 billing code search. Inclusion criteria were (1) diagnosis of SRNS, with a minimum of 6 months follow-up from initial diagnosis; (2) age 1–18 years at the time of diagnosis; and (3) treatment with either cyclosporine A and/or tacrolimus. Exclusion criteria were (1) steroid dependent and/or frequently relapsing NS, (2) late-onset steroid resistance, or (3) incomplete medical records. Definitions of steroid response, remission, and relapse were based upon the 2012 KDIGO guidelines (11).

### Data Collection

Data collected from the medical records of children meeting inclusion criteria included basic demographic data (age, gender, and race), clinical features at diagnosis (presence of hematuria, hypertension, serum creatinine, and urine protein/creatinine ratio), histopathology, and growth parameters (weight and height). Longitudinal follow-up data were also collected and included number of relapses/remissions, complications of disease/treatment [infections requiring hospital admission, development of diabetes mellitus, development of hypertension, thrombosis/stroke, and episodes of acute kidney injury (AKI)], and estimated GFR (eGFR). AKI was defined as a 0.3 mg/dL rise in the serum creatinine within a 48-h period per the AKI network criteria (19). eGFR was calculated using the original bedside Schwartz equation and the modified Schwartz equation when serum creatinine was measured using isotope dilution mass spectrometry (IDMS) (20, 21). End follow-up occurred at the completion of the study period or when the patient progressed to ESRD (transplantation or dialysis).

### Statistical Analysis

Baseline results were expressed as means with ranges and percentages. Given the small patient sample size normality was not assumed and all statistics were performed non-parametrically. Due to the small sample size, all patients were included in the final statistical analysis regardless of their initial response to calcineurin inhibition. Statistical tests utilized include Pearson chi-squared, Mann–Whitney *U* test, Pearson correlation, Spearman rank correlation, and Kaplan–Meier survival analysis. All statistical analyses were performed with the SPSS software suite (version 22.0). The research design and statistical analysis was approved by University Hospitals/Case Western Reserve University Institutional Review Board.

## Results

A total of 34 patients were identified of which 16 met all inclusion criteria. Of the 18 excluded patients, 14 had either late-onset steroid resistance or steroid dependence, 2 patients were not treated with CNIs, and 2 patients’ records were unavailable. Mean age at onset of SRNS was 6.9 years (1.7–13 years) and mean duration of follow-up was 6.6 years (range 0.6–17.6 years). Seven of the 16 children were male (43.8%). Nine of the children were African-American (56.2%), four children were Caucasian (25%), and three children were Hispanic (18.8%). All patients underwent biopsy soon after diagnosis of SRNS with eight patients having focal segmental glomerular sclerosis (50%), four with mesangial proliferative glomerulonephritis (25%), three with IgM nephropathy (18.8%), and one patient with minimal change disease (6.2%). The majority of patients were treated with cyclosporine A (10 patients, 62.5%) with only 2 patients (12.5%) receiving tacrolimus, whereas 4 patients (25%) were treated with both medications during the study period. Demographic and clinical features of the study cohort are summarized in Table 1.

**Table 1:** **Study population baseline characteristics**.

Gender	Male: 7 (44%)
	Female: 9 (56%)
Mean age at onset (years)	6.9 (1.7–13.9)
Histopathology	FSGS: 8 (50%)
	MPGN: 4 (25%)
	IgM nephropathy: 3 (19%)
	Minimal change: 1 (6%)
Ethnicity	African-American: 9 (56%)
	Caucasian: 4 (25%)
	Hispanic: 3 (19%)
Medication	Cyclosporine A: 10 (63%)
	Tacrolimus: 2 (12%)
	Both: 4 (25%)

Thirteen of the 16 patients (81.3%) achieved remission with CNI therapy. The three patients who failed to achieve initial remission all progressed to ESRD during the length of follow-up (7, 11 months, and ~5 years). Among patients achieving initial remission, relapses were common with a mean of 3.4 relapses (0.5 relapses/year). Complications that arose during the course of treatment included infections requiring admission (three patients), steroid-induced cataracts (one patient), venous/arterial thrombosis (three patients), and one patient who suffered posterior reversible encephalopathy syndrome. AKI was also very common with 13/16 patients (81.3%) having at least one episode of AKI. The mean number of AKI episodes was 2.1 ± 1.5 among all 16 patients in the study, which corresponds to a mean 0.7 ± 1.1 AKI episode per patient year of follow-up. When restricted to only those patients achieving remission with CNI therapy (*n* = 13) the mean number of AKI episodes per patient year was 0.34 ± 0.3 episodes. Reversibility to baseline creatinine was seen in 71% of AKI episodes with the remaining 29% adopting a new baseline.

**Figure 1:**
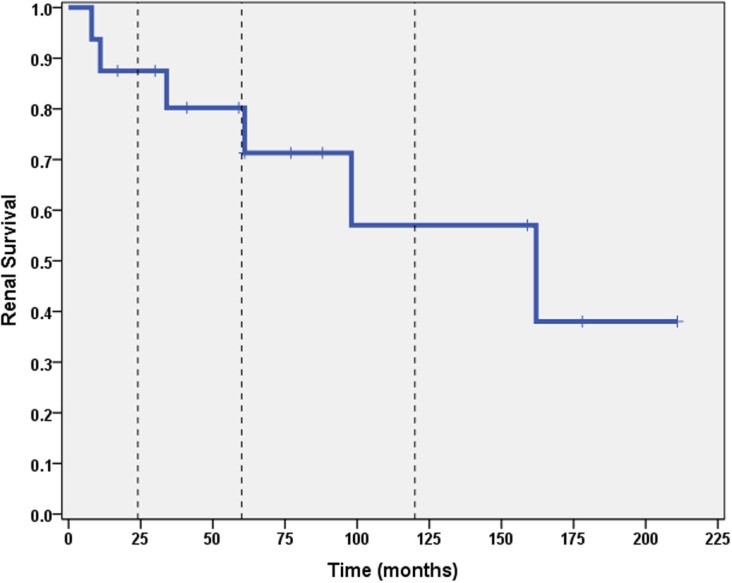
**Kaplan–Meier renal survival curve for all subjects**. Vertical dashed lines represent time points of 2, 5, and 10 years for both CNI responders and CNI non-responders.

**Figure 2:**
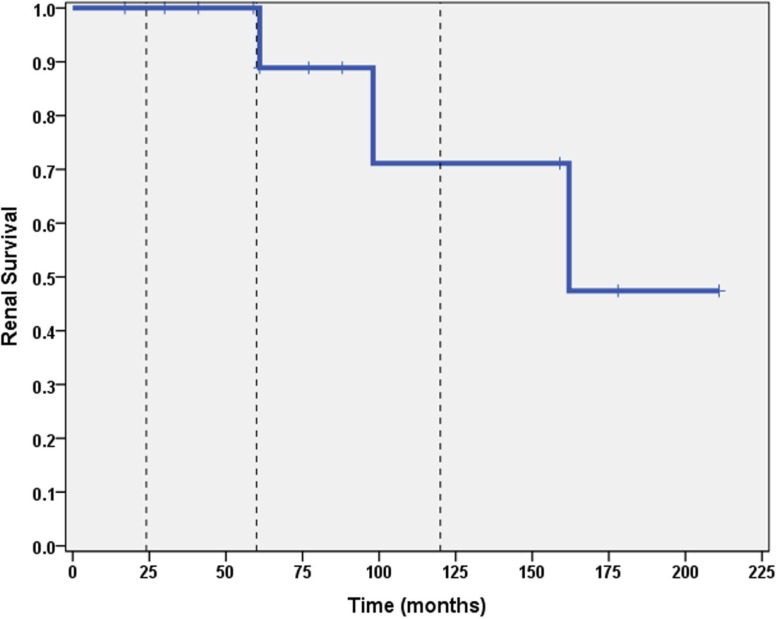
**Kaplan–Meier renal survival curve for calcineurin inhibitor responders**. Vertical dashed lines represent time points of 2, 5, and 10 years for subjects who initially responded to CNI inhibitors.

A total of 6 out of 16 patients (37.5%) developed ESRD during the study period: 3/3 (100%) of CNI non-responders and 3/13 (23%) of CNI responders. Table 2 summarizes the demographic and clinical features results based on renal outcome (ESRD vs. non-ESRD). The only two significant associations with ESRD in the study population were non-Caucasian race (*p* = 0.024), and the mean number of AKI episodes per patient year of follow-up (*p* = 0.031). No other significant associations with ESRD were identified in the study population with respect to age, gender, histopathology, medication, or number of remissions. Finally, eGFR was inversely associated with a higher mean number of AKI episodes (*p* = 0.022, 95% confidence interval = −28.5 to −2.5 mL/min/1.73 m^2^). No other associations with lower final eGFR were identified. Kaplan–Meier renal survival analysis was performed and demonstrated survival rates of 87, 71, and 57% at 2, 5, and 10 years, respectively (see Figure 1). When CNI non-responders were eliminated from the statistical analysis, the only significant correlation was AKI episodes per patient year of follow-up with lower eGFR (*p* = 0.03). Kaplan–Meier renal survival analysis for CNI responders demonstrated renal survival rates of 88, 80, and 57% at 2, 5, and 10 years, respectively (see Figure 2).

**Table 2:** **Study demographics and results by renal outcome**.

	Non-ESRD (*n* = 10)	ESRD (*n* = 6)	*p*-Value
Gender (% male)	4 (40%)	3 (50%)	0.696
Mean age at onset (years)	6.1 (1.7–13.2)	8.5 (1.7–13.9)	0.301
Histopathology	FSGS: 5 (50%)	FSGS: 3 (50%)	0.828
	MPGN: 3 (30%)	MPGN: 1 (17%)	
	IgM nephropathy: 1 (10%)	IgM nephropathy: 2 (33%)	
	Minimal change: 1 (10%)		
Ethnicity	African-American: 6 (50%)	African-American: 3 (50%)	**0.024**
	Caucasian: 4 (40%)	Hispanic: 3 (50%)	
Medication	Cyclosporine A: 8 (80%)	Cyclosporine A: 2 (33%)	0.147
	Tacrolimus: 1 (10%)	Tacrolimus: 1 (17%)	
	Both: 1 (10%)	Both medications: 3 (50%)	
Mean follow-up duration (months)	92 (17–211)	61 (7–162)	0.278
Mean number of relapses	3.2	3.8	0.428
Mean number of AKI episodes	1.5	3.3	0.073
Mean number of AKI episodes (per patient year of follow-up)	0.27	1.5	**0.031**

*Red bold text indicates statistical significance*.

## Discussion

Steroid-resistant nephrotic syndrome remains a challenging clinical entity and many patients still struggle to achieve remission despite newer therapies. Failure to achieve remission with steroids was historically associated with rates of progression to ESRD >50% within a 5-year period (9, 10). Since the advent of CNIs (and other therapies), there has been a paucity of long-term outcome data regarding progression to ESRD. This study provides further evidence regarding the long-term outcomes of SRNS. A significant percentage (18.8%) failed to respond to CNIs, all of whom progressed to ESRD. Among those initially responding to CNIs, 23% (3/13) still progressed to ESRD. Although renal survival at 1 and 5 years was still fairly good (>80%), the 10-year renal survival of that group was 57%, demonstrating the importance of following these long-term clinical course.

A growing body of evidence in adult nephrology literature that suggests the recurrent episodes of AKI may be associated with worse chronic kidney disease (CKD) (22–24). Several studies in pediatric ICU settings have demonstrated development of CKD after AKI in pediatric patients (25, 26). Given that patients with SRNS treated with CNIs, which may also be treated with angiotensin converting enzyme inhibitors or angiotensin receptor blockers, we hypothesized that this population is at high risk for developing AKI.

Our study did, in fact, confirm that AKI is a common occurrence among SRNS patients, with more than 80% of patients suffering an episode of AKI during their follow-up period and most patients having more than one episode of AKI. Exact etiology of these AKI episodes could not be determined retrospectively; however, the majority of AKI episodes occurred during time of relapse. Prior studies examining complications of nephrotic syndrome (not specifically SRNS) demonstrate that ~10% of all admissions for nephrotic syndrome are associated with AKI (27). Furthermore, we identified a significant association between AKI episodes and progression to ESRD. We hypothesize that these episodes of AKI lead to cumulative damage that increases the risk of progression to ESRD. This finding highlights the importance of rapidly identifying and managing even mild AKI in this population.

Several other studies have examined the outcome of SRNS but with important limitations. Otukesh et al. (15) examined 59 children with early steroid resistance and found a renal survival rate of 56% at 10 years; however, the study included both early and late steroid resistance and therapy with cyclophosphamide and mycophenolate mofetil in addition to cyclosporine A. Gellermann et al. (28) demonstrated effectiveness in achieving sustained remission using a combination of cyclosporine and mycophenolate mofetil in early SRNS secondary to FSGS with a 100% renal survival rate in their cohort. However, the study only included Caucasian children and at enrollment all patients were in remission via standard steroids, high dose methylprednisolone ± addition of cyclosporine if proteinuria did not improve. It is unclear how many of the given patients may have responded to the high dose steroids without the addition of cyclosporine and the inclusion of only a single ethnicity makes it difficult to extrapolate the results to other populations. Similarly, Hamasaki et al. (18) performed a prospective 5-year study examining SRNS and cyclosporine and found a renal survival rate of 94.3%. However, their study also was of a single ethnicity and the majority of the enrolled patients had minimal change disease with only 20% having FSGS. In addition, the shorter follow-up time compared to our study could have precluded identifying children who would still enter ESRD but at a later timepoint. Finally, in the largest series published to date, Zagury et al. (17) examined the outcomes of SRNS in their population within Brazil of 114 patients with early steroid resistance treated with cyclosporine A. They found a renal survival rate of 58.4% at 10 years and identified FSGS and cyclosporine resistance as predictors of progression to ESRD. These study, however, did not examine episodes of AKI.

As with several of the published studies, our study had important limitations, including its retrospective, single center nature, and the small sample size. In addition, the majority of our patients in this study were treated with cyclosporine A, so data on tacrolimus were limited. Target CNI levels varied throughout the study follow-up period at the discretion of the treating physician. Trough levels were examined; however, they were highly variable due to numerous factors including improperly timed collection, patient non-compliance, and impact of illness. Due to this variation analysis of the impact of CNI trough levels was unable to be performed. Finally, although we identified an association between AKI and progression to ESRD, it is possible that the increased number of AKI episodes may reflect more severe baseline disease that would be more likely to progress to ESRD, rather than being a causative factor of ESRD. Severity of baseline disease could be further assessed by FSGS histopathology; however, these data were unavailable in our study due to many biopsies occurring prior to the development of the classification scheme used to identify FSGS histological variants. Further studies examining the role of FSGS histopathology, AKI, and CKD progression are needed to address this concern.

In conclusion, this study demonstrates that treatment with CNIs can be effective in achieving sustained remission for idiopathic SRNS. Although short-term prognosis (<5 years) was generally good, our study found that long-term renal survival was less favorable, with 43% of CNI-responsive patients progressing to ESRD at 10 years. These findings are consistent with previously published data, which suggest that, although newer therapies are slowing the rate of progression, close to 50% will still reach ESRD during childhood/early adulthood (16–18). Notably, our study found that increased episodes of AKI were associated with increased risk of ESRD. While we were unable to establish causality, these findings provide, for the first time, support for a relationship between repeated AKI and progression to ESRD in SRNS A larger, multi-center prospective study would be necessary to further define long-term outcomes of a diverse population of children with SRNS responsive to CNIs to and determine the role, if any, that episodes of AKI may play in the progression to ESRD. The possibility of long-term adverse effects of AKI episodes in patients with SRNS emphasizes the importance of AKI prevention in this population. Future treatment guidelines should stress the importance of AKI prevention through avoidance of dehydration, nephrotoxic medications, and prompt treatment of infections and relapse episodes.

## Author Contributions

NB performed the initial chart review, data collection, statistical analysis, and manuscript authorship. KD assisted with the study design, identification of study participants, interpretation of study results, and manuscript authorship.

## Conflict of Interest Statement

The authors declare that the research was conducted in the absence of any commercial or financial relationships that could be construed as a potential conflict of interest.
